# Design and Evaluation of ScanCap: A Low-Cost, Reusable Tethered Capsule Endoscope with Blue-Green Illumination Imaging for Unsedated Screening and Early Detection of Barrett’s Esophagus

**DOI:** 10.3390/bioengineering11060557

**Published:** 2024-05-31

**Authors:** Cheima Hicheri, Ahad M. Azimuddin, Alex Kortum, Joseph Bailey, Yubo Tang, Richard A. Schwarz, Daniel Rosen, Shilpa Jain, Nabil M. Mansour, Shawn Groth, Shaleen Vasavada, Ashwin Rao, Adrianna Maliga, Leslie Gallego, Jennifer Carns, Sharmila Anandasabapathy, Rebecca Richards-Kortum

**Affiliations:** 1 Department of Bioengineering, Rice University, Houston, TX 77030, USA; cheima.hicheri@rice.edu (C.H.); schwarzr@rice.edu (R.A.S.);; 2 Houston Methodist Hospital, Houston, TX 77030, USA; aazimuddin@houstonmethodist.org; 3 Texas A&M School of Medicine, Houston, TX 77030, USA; 4 Rice360 Institute for Global Health Technologies, Rice University, Houston, TX 77030, USA; 5 Baylor College of Medicine, Houston, TX 77030, USAleslie.gallego@bcm.edu (L.G.);

**Keywords:** Barrett’s esophagus, narrow-band imaging, digital chromoendoscopy, capsule endoscopy, low-resource settings, cancer detection, esophageal cancer, cancer screening, global health

## Abstract

Esophageal carcinoma is the sixth-leading cause of cancer death worldwide. A precursor to esophageal adenocarcinoma (EAC) is Barrett’s Esophagus (BE). Early-stage diagnosis and treatment of esophageal neoplasia (Barrett’s with high-grade dysplasia/intramucosal cancer) increase the five-year survival rate from 10% to 98%. BE is a global challenge; however, current endoscopes for early BE detection are costly and require extensive infrastructure for patient examination and sedation. We describe the design and evaluation of the first prototype of ScanCap, a high-resolution optical endoscopy system with a reusable, low-cost tethered capsule, designed to provide high-definition, blue-green illumination imaging for the early detection of BE in unsedated patients. The tethered capsule (12.8 mm diameter, 35.5 mm length) contains a color camera and rotating mirror and is designed to be swallowed; images are collected as the capsule is retracted manually via the tether. The tether provides electrical power and illumination at wavelengths of 415 nm and 565 nm and transmits data from the camera to a tablet. The ScanCap prototype capsule was used to image the oral mucosa in normal volunteers and ex vivo esophageal resections; images were compared to those obtained using an Olympus CV-180 endoscope. Images of superficial capillaries in intact oral mucosa were clearly visible in ScanCap images. Diagnostically relevant features of BE, including irregular Z-lines, distorted mucosa, and dilated vasculature, were clearly visible in ScanCap images of ex vivo esophageal specimens.

## 1. Introduction

Esophageal cancer is the sixth-leading cause of cancer death and the eighth-most-common cancer worldwide [1,2]. The incidence of esophageal adenocarcinoma (EAC), a subtype of esophageal cancer, has increased worldwide in recent decades [3,4]. The asymptomatic nature of EAC results in most cases being found incidentally and at a late stage; only 12.5% of EACs are found at a stage that allows for endoscopic resection [5]. Despite improvements in management and treatment, five-year survival rates for this malignancy are only approximately 10% when diagnosed at a late stage and have remained at only approximately 40% even after esophagectomy [2,6,7]. The poor survival rate after late-stage diagnosis emphasizes the need for early detection. The only known precursor to EAC is a columnar metaplasia, known as Barrett’s Esophagus (BE) [8,9]. Surveilling and treating BE are crucial to the prevention of EAC. When EAC is detected early via adequate screening for BE, the five-year survival rates can exceed 98% [10]. Unfortunately, most patients diagnosed with EAC were not known to have BE beforehand [11]. Meanwhile, 12% of EAC patients had a prior diagnosis of BE, and BE was found in 57% of patients at the time of EAC diagnosis, meaning a large number of patients face morbidity that was otherwise preventable by effective BE screening [12]. Additional studies have shown that patients who have their cancers diagnosed through BE surveillance programs have more favorable outcomes than patients with EAC discovered when they present with cancer symptoms [13]; thus, there is a clear benefit to identifying patients with BE early and enrolling them in surveillance. Current American College of Gastroenterology guidelines for BE screening are expensive to implement and inadequate in the face of the increasing incidence of EAC [14,15]. Screening involves direct upper endoscopy to visualize the presence of dysplasia at the gastroesophageal junction (GEJ), with at least eight biopsy samples to be evaluated by a trained pathologist with gastrointestinal (GI) expertise. This procedure is technically complex and invasive, requires costly equipment (endoscopy tower and scope), and requires skilled gastroenterology, anesthesiology, and pathology personnel [16]. As a result, comprehensive screening for BE is impossible at many hospitals in low- and middle-income countries (LMICs), leading to a lack of organized and comprehensive esophageal cancer screening programs [17,18,19]. To maintain cost-effectiveness, available screening is limited to those with chronic and/or frequent symptoms of gastroesophageal reflux disease (GERD) who also have ≥3 risk factors: male sex, age > 50 years, Caucasian race, obesity, tobacco, and a confirmed family history of BE or EAC (in a first-degree relative), thus missing many candidates for intervention in LMICs [14,20,21]. Heterogeneity in incidence and risk factors and ambiguity in presentation and diagnosis produce additional challenges [17]. As a result, 85% of all esophageal cancer deaths—both EAC and esophageal squamous cell carcinoma (ESCC)—occur in LMICs across Central Asia and the eastern coast of Africa [22]. Modified screening guidelines involving cluster screening of high-risk populations across China have been proposed, yet a lack of sensitivity, specificity, and affordability has limited their effectiveness [19]. In conjunction with developing screening guidelines, alternative screening technologies have emerged as a potential mechanism for cost reduction [11,18,23,24]. Trans-nasal endoscopy (TNE) uses a thin endoscope that can be introduced through the nose, a procedure performed without sedation. However, TNE generally still requires significant training [25], and the nasal passage is uncomfortable to many patients. Cytosponge (Medtronic, Dublin, Ireland) is a low-cost cell sampling device that can be administered easily in a primary care setting, with no advanced training required. However, recent data from a multi-center US trial showed that the overall sensitivity, specificity, and accuracy of the Cytosponge were lower than desired for screening [26,27,28]. Breath testing via the detection of volatile organic compounds in exhaled air has been developed, with a sensitivity and specificity of 91% and 74%, respectively, but with limited validation or generalizability in clinical care settings [29]. Esophageal video capsule endoscopy (VCE) is less invasive, more cost effective [30], better tolerated [31], and preferred by patients [32,33] when compared to standard endoscopy. Since the approval of VCE to detect lesions in the GI tract, a series of pill endoscopes have emerged. Traditional pill endoscopes include the PillCam ESO2 (Medtronic, Dublin, Ireland), PillCam ESO3 (Medtronic, Dublin, Ireland), and CapsoCam Plus (CapsoVision, Saratoga, CA, USA) [34,35]. Although these capsules are primarily intended to analyze only the small bowel and colon [36,37], VCE has been studied for BE screening. However, its widespread use has been limited by lower image quality and accuracy than standard upper endoscopy of the upper GI tract [38]. Given the limitations of existing screening tools and the high mortality of EAC when diagnosed late, there is a substantial need for a cost-effective screening modality that non-expert clinicians can use without patient sedation to screen for BE. Here, we introduce ScanCap (Martigues, France), a low-cost, reusable, tethered capsule endoscope, incorporating blue-green illumination (BGI) for non-sedated screening and early detection of BE. BGI is an imaging technique that allows for the assessment of abnormal vasculature using a narrowed light spectrum. The narrowed illumination band utilizes blue and green wavelengths that match the absorption spectrum of hemoglobin, allowing for greater contrast to visualize abnormal vasculature patterns on mucosal and submucosal surfaces. Under blue light, superficial capillaries appear brown, while under green light, submucosal capillaries appear cyan [39]. This method is one of only two imaging modalities (the other being confocal laser endoscopy) satisfying the American Society for Gastrointestinal Endoscopy Preservation and Incorporation of Valuable Endoscopic Innovations (ASGE PIVI) negative predictive value (NPV > 98%) for ‘optical biopsy’ in BE [40]. This feature sharpens the visual field while inspecting the squamocolumnar junction, helping detect BE and dysplasia [39]. This novel capsule combines the resolution and contrast of gold-standard endoscopy with the cost and ease of use of capsule technologies. Here, we demonstrate the construction of the capsule and its associated illumination module, the implementation of BGI, and the evaluation of capsule imaging performance in resected esophageal cancer specimens and the oral mucosa of healthy volunteers.

## 2. Materials and Methods

### 2.1. ScanCap System Design and Workflow

**Figure 1:**
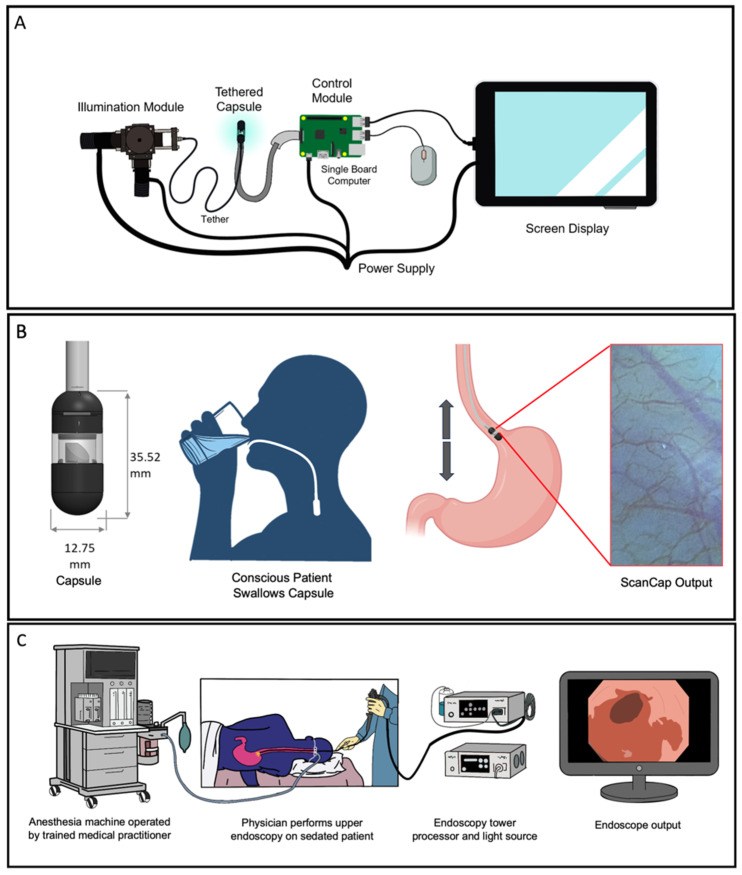
Overview of current ScanCap system and assessment versus the current standard of care. (**A**) The complete ScanCap system consists of a tethered capsule and a control system comprising an LED-based illumination module, a control module with a single board computer and power supply, and a screen display. (**B**) Proposed procedure to image the esophagus using the ScanCap endoscopy capsule. Cross-sectional overview of ScanCap; a conscious, non-sedated patient swallows the tethered capsule. Forward motion of the capsule is propelled via the natural peristaltic forces of the esophagus; the capsule is withdrawn manually using the tether. (**C**) Standard endoscopy procedure to image the esophagus. The patient is sedated while an endoscope is introduced into the upper gastrointestinal tract.

Figure 1 illustrates the components of the ScanCap capsule endoscopy system (Figure 1A) and compares the clinical workflow for the detection of BE envisioned with ScanCap (Figure 1B) to the current standard of care (Figure 1C). As shown in Figure 1A, ScanCap is a low-cost high-resolution optical endoscopy system consisting of (1) a capsule, and (2) a control system comprising an LED-based illumination module, a control module including a single board computer and power supply, and a screen display. ScanCap is designed to provide high-definition BGI imaging of the esophagus. The tethered biocompatible capsule (12.8 mm diameter, 35.5 mm length) contains a color camera with a 1920 × 1080 image sensor resolution and a rotating mirror. It is designed to be swallowed by a conscious patient and propelled to the GEJ via natural peristaltic forces generated by esophageal musculature (Figure 1B). The tether consists of tubing that houses cables to provide electrical power and illumination at wavelengths of 415 nm and 565 nm. As the capsule is withdrawn manually, circumferential images are collected and stitched together to form a two-dimensional map of the esophagus. The current specifications for the ScanCap capsule compared to a standard-of-care imaging modality (Olympus GIF-HQ190 Endoscope, Olympus America Inc., Center Valley, PA, USA) are shown in Table 1. ScanCap is designed to be swallowed, with a tether length suitable to reach the GEJ, a high spatial resolution, and BGI integration.

### 2.2. ScanCap Capsule Design

**Figure 2:**
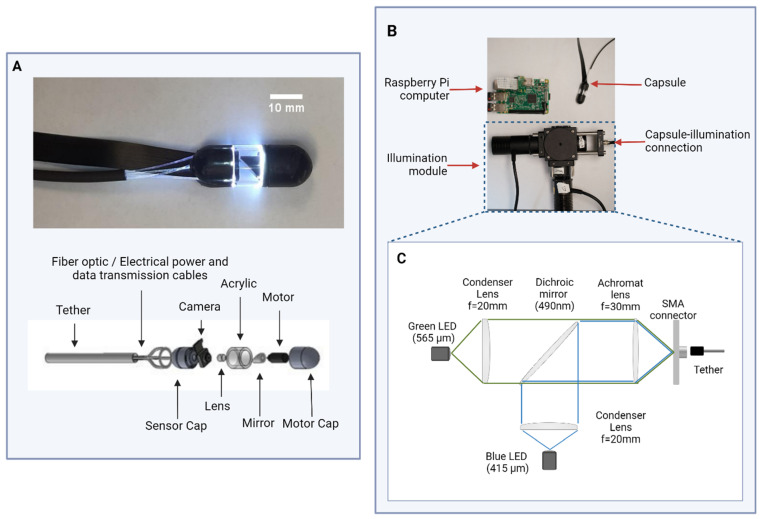
Schema of the ScanCap system and photographs of prototype. (**A**) Photograph (top) and schematic (bottom) of prototype ScanCap capsule, including tether, optical fibers that deliver blue and green light from the illumination module, 3D-printed sensor cap that holds Raspberry PiCam and lens, acrylic wall, mirror, micromotor, and 3D-printed motor cap. (**B**) Photograph of ScanCap capsule and control system modules, including the illumination and control modules. (**C**) Schematic diagram of illumination module.

Figure 2A shows a schematic diagram and photograph of the tethered capsule. The capsule consists of two 3D-printed biocompatible caps printed with Formlabs Biomed Black resin attached to an 8.8 mm length section of clear acrylic tubing. The distal cap of the capsule endoscope contains a miniature rotational stepper motor (Faulhaber DM 0620, Faulhaber, Clearwater, FL, USA) attached to a 45-degree-angle mirror. The proximal cap of the capsule endoscope contains a color camera (Raspberry PiCam camera module, Raspberry Pi Foundation, Cambridge, UK) and an achromatic doublet focusing lens (d = 4 mm, f = 10 mm, MgF Coated). Electrical power and data transmission to the motor are provided through the tether, which is enclosed in polyurethane tubing. In addition, the tether contains seven 0.5 mm diameter optical fibers (Edmund Optics #53-833, Edmund Optics Inc., Barrington, NJ, USA) that direct illumination light to the proximal wall of the acrylic tubing to provide uniform illumination. Presently, video data are transmitted from the camera to the control module via an Arducam extension cable (Arducam B0186, Arducam, Nanjing, China), with plans to integrate this video cabling within the tether tubing in future iterations.

The capsule endoscope is designed to image tissue in contact with the outer surface of the acrylic tubing. Illumination light is directed to the tissue via internal reflection. Light reflected from the tissue surface is directed by the mirror onto the camera, which captures a 12 mm × 7 mm field of view (FOV). The motor rotates the mirror at a rotational speed of up to 30 rpm, allowing the camera to sequentially capture high-definition images of the full 360-degree FOV. To avoid motion artifacts from the rotating mirror, the camera and mirror are synced so that every third image is captured while the motor is stationary and saved for processing. The camera operates at a frame rate up to 30 frames per second (fps) (based on the rotational speed of the mirror), resulting in an image acquisition time of up to 10 fps. The exposure time is maximized to match the duration of time that the motor is stationary. As the capsule is withdrawn, a series of images are captured to produce a map of the interior esophageal surface. The system is intended to image a 5 cm portion of the lower esophagus (the location where BE is formed), beginning at 1 cm below and ending at 4 cm above the GEJ. As each image corresponds to a 12 × 7 mm^2^ area of tissue, a minimum of 100 images are necessary to fully map this portion of the esophagus; allowing for a 50% overlap of each image to register and stitch the images together, imaging can be completed in less than 20 s, enabling a procedure time of less than one minute.

### 2.3. ScanCap Control System Design

Figure 2B shows the ScanCap control system, which consists of an illumination module, a control module, and a tablet display. The ScanCap illumination module (Figure 2C) provides illumination for BGI. It utilizes two LED light sources: a blue LED (Thorlabs M415L4, Thorlabs Inc., Newton, NJ, USA) with a center wavelength of 415 nm and a green LED (Thorlabs M565L3, Thorlabs Inc., Newton, NJ, USA) centered at a wavelength of 565 nm. Each LED is collimated using a 25 mm diameter aspheric condenser lens (Thorlabs ACL2520U-A, Thorlabs Inc., Newton, NJ, USA) with a 20 mm focal length and NA = 0.60. The light sources are positioned perpendicular to each other on a cage cube, and illumination is combined using a dichroic mirror (Thorlabs DMLP490R, Thorlabs Inc., Newton, NJ, USA) positioned at a 45-degree angle and focused into the optical fibers using a 25.4 mm diameter visible achromat lens (Thorlabs AC254-030-A, Thorlabs Inc., Newton, NJ, USA) with a focal length of 30 mm. The capsule is then connected to the illumination module via an SMA connector.

The control module comprises a power supply and a Raspberry Pi 4 Computer (Raspberry Pi Foundation, Cambridge, UK). This module connects to ScanCap via the Arducam extender. The Raspberry Pi 4 can be connected to any commercial tablet display. A Python (version 2.7) script run on the Pi Computer synchronizes the movement of the motor and video capture from the ScanCap PiCamera.

### 2.4. Motor Rotation

**Figure 3:**
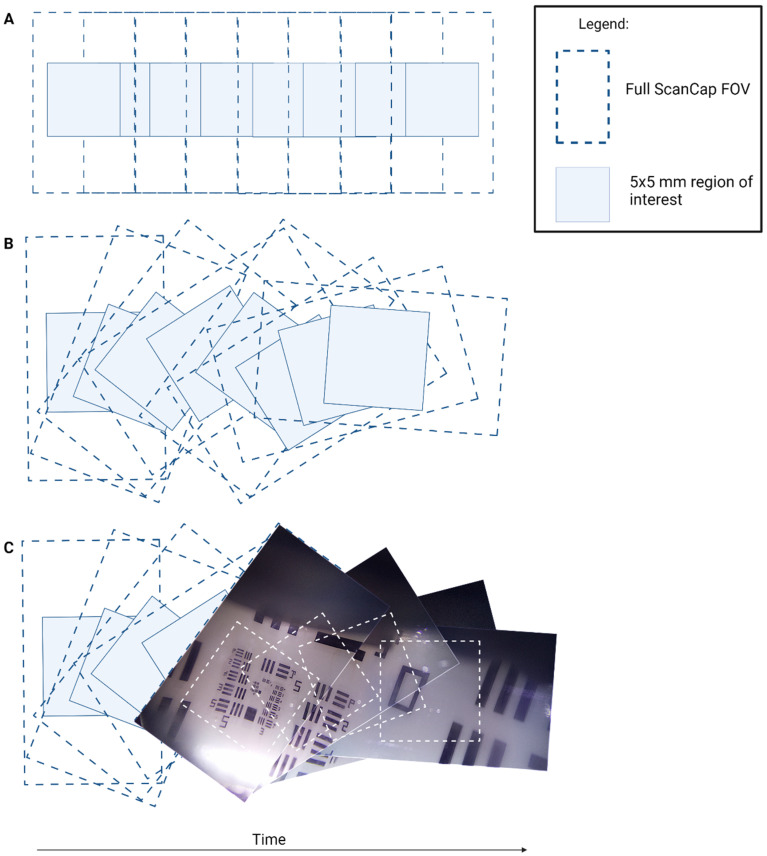
Diagram of image sequence acquisition. (**A**) Hypothetical image capture sequence if the mirror and the camera were rotated together. (**B**) Actual ScanCap image sequence with rotating mirror and stationary camera. (**C**) Image sequence taken with ScanCap with motor rotating the mirror. In each diagram, the full ScanCap field of view (FOV) (12 × 7 mm^2^) and the smaller region of interest (5 × 5 mm^2^) are depicted. The full ScanCap FOV is utilized for mosaicking purposes and enables full coverage of the center 5 × 5 mm^2^ region of interest.

The ScanCap stepper motor is housed inside the capsule on the opposite end of the camera and rotates the mirror, allowing for a 360-degree view of the surrounding tissue. Figure 3 shows the image sequence observed when images are captured with the motor running. Figure 3A shows the hypothetical image sequence that would be observed in the case where the mirror and camera rotate together. However, in the ScanCap capsule, the camera remains stationary while the mirror rotates, leading to the sequence pattern shown in Figure 3B,C. Using positional information from the motor, the captured images are then stitched back together, as shown in Figure 3C.

### 2.5. Performance Evaluation

The resolution and FOV of ScanCap were evaluated by collecting images of a 1951 United States Air Force (USAF) resolution test target. The USAF target was placed on a flat surface. ScanCap was then placed over this target, with the acrylic side wall in direct contact. Water was applied on the resolution target for index matching.

To evaluate the performance of the ScanCap capsule while the motor is operational, a 1951 USAF resolution target was wrapped around the capsule in contact with its surface. Similarly, water was applied between the target and the acrylic for index matching. The motor steps and captures an image every 18 degrees. A total of 20 images are captured in one full rotation. The USAF resolution target images acquired are then stitched back together.

To measure the depth of focus (DOF), the ScanCap capsule was placed in contact with a fixed USAF resolution target on a moving stage. The capsule was then incrementally moved away from the target until it was no longer able to resolve group 1 element 1 (250 µm) of the USAF resolution target. Illumination uniformity was assessed across a single captured image as well as across the 360-degree view of the capsule. Pixel intensity was measured as a function of angular degree to assess the illumination uniformity.

ScanCap was used to collect in vivo images of oral mucosa from healthy volunteers. The protocol was reviewed and approved by the Institutional Review Board of Rice University (Protocol IRB-FY2021-71); subjects provided written informed consent. The ScanCap capsule was placed against the inner lining of the oral mucosa, and images were collected. The capsule was then rolled or translated across the mucosa to capture multiple images. Following imaging with the capsule, the oral mucosa was also imaged with a conventional endoscope (Olympus EVIS/EXERA II CV-180 and GIF-H180 Scope, Olympus America Inc., Center Valley, PA, USA) for comparison. Prior to imaging normal volunteers’ oral mucosa, the endoscope’s resolution was evaluated using a USAF resolution target.

To evaluate the ability of the capsule to detect the characteristic features of BE, ScanCap was used to collect images from resected human esophageal specimens. The protocol was reviewed and approved the by the Institutional Review Boards of Baylor College of Medicine and Rice University (IRB protocols H-41066 and 2017-514, respectively). Subjects scheduled to undergo an esophagectomy procedure provided written informed consent. The capsule was placed in contact with the resected esophageal tissue and translated across the surface near the GEJ, and images were captured.

ScanCap tissue images were processed using a contrast-enhancing and image-sharpening algorithm created via Labview (Labview 2012, National Instruments, Austin, TX, USA). This feature utilized a series of filters to enhance edges within the image to produce a sharpened image. Details regarding this algorithm can be found in [42].

### 2.6. Safety Evaluation

Three prototype ScanCap capsules consisting of the capsule outer components (Sensor Cap, Acrylic, Motor Cap and biocompatible tubing used as the tether) sealed together with medical-grade epoxy (EPO-TEK MED-301, Epoxy Technology, Cranston, RI, USA) were used to validate the integrity of the capsule seal. These prototypes were then submerged in a hydrochloric acid–potassium chloride solution buffer at pH 1.4 to mimic gastric contents. After 24 h of submersion, capsules were removed from the solution and examined by visual inspection under a dissecting microscope. The seal integrity was also evaluated by blowing air into the capsule through the polyurethane tether using a compressed air pump and measuring any changes in pressure within the capsule using a small commercial pressure gauge (Molten USA PG, Molten, Reno, NV, USA).

To evaluate the potential for the ScanCap capsule surface to damage the esophageal surface during introduction and withdrawal, we repeatedly introduced a similar capsule prototype to the one used for the seal integrity testing into an intact resected porcine esophagus obtained from an abattoir, and an experienced endoscopist examined the inner surface of the tissue for abrasions using an Olympus CV-180 (Olympus America Inc., Center Valley, PA, USA) endoscope, before and after passing the prototype ScanCap capsule to the GEJ. The outside surface of the capsule was visually inspected for any irregularities.

## 3. Results

### 3.1. Performance Evaluation Results

**Figure 4:**
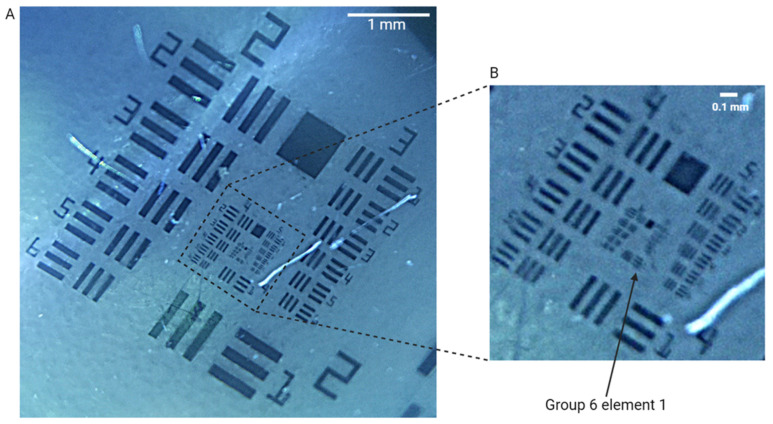
(**A**) ScanCap image of 1951 USAF resolution test target showing a 5 mm × 5 mm area of the full field of view (FOV). (**B**) Zoomed-in picture of the United States Air Force (USAF) standard resolution target showing that ScanCap can resolve group six, element one (7.8 µm).

Figure 4 shows an image of a 1951 USAF resolution test target obtained with ScanCap; group 6 element 1 is clearly resolved, corresponding to a resolution of 7.8 µm. The DOF was assessed to be 3.5 mm. The system provides illumination uniformity, from both input sources, with less than 10% intensity deviation across the 360-degree view as well as across a single captured image frame.

### 3.2. Motor Results

**Figure 5:**
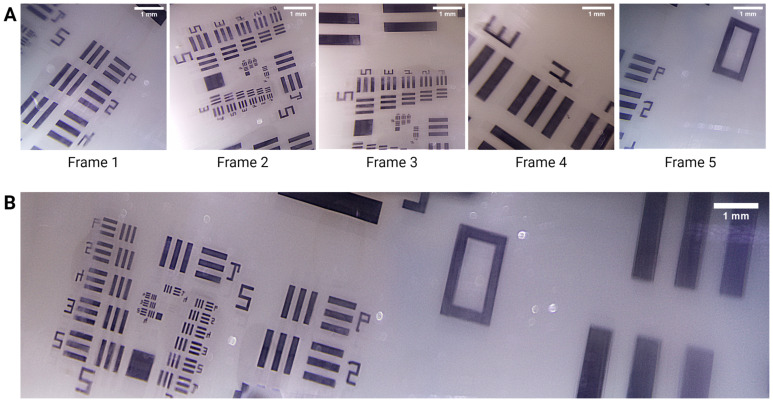
(**A**) Representative single frames acquired while the motor rotated. Each image shows a 5 mm × 5 mm segment of the 12 × 7 mm^2^ field of view (FOV). (**B**) Stitched image of the frames captured in (**A**).

Figure 5 shows the resulting images from a USAF resolution target captured with ScanCap while the motor is operational. Five consecutive frames (Figure 5A) were stitched together to form the reconstructed image in Figure 5B.

**Figure 6:**
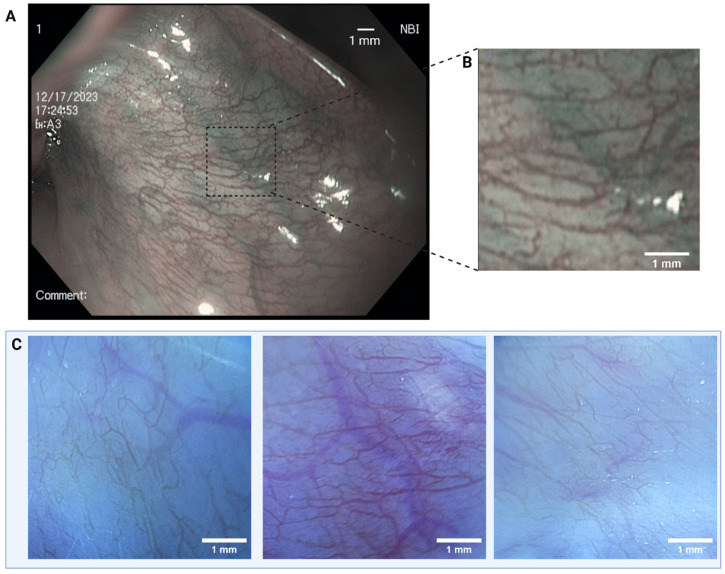
(**A**) Image of buccal mucosa (lower lip) obtained using an Olympus CV-180 endoscope with narrow-band imaging. (**B**) A 5 mm × 5 mm zoomed-in image from a representative area of the endoscopic capture. (**C**) Images of human buccal mucosa (lower lip) obtained with ScanCap. The ScanCap field of view (FOV) is cropped to show a 5 mm × 5 mm segment of the 12 × 7 mm^2^ FOV.

Figure 6 shows images of the oral mucosa of a healthy volunteer in vivo obtained using an Olympus Exera CV-180 endoscope (Figure 6A) as well as images from the same volunteer’s oral mucosa obtained with ScanCap (Figure 6C). Each ScanCap image represents a 5 × 5 mm^2^ segment of the 12 × 7 mm^2^ FOV, and all images were taken with the same illumination parameters. Figure 6A shows an image of the oral mucosa in a normal volunteer taken with a standard commercial endoscope using narrow-band imaging (NBI). The resolution of the endoscope in this configuration was measured to be 44.2 µm. Figure 6B depicts the 5 mm × 5 mm FOV that is captured by ScanCap. Images obtained with ScanCap clearly delineate microvasculature and demonstrate that the same diagnostically relevant features can be visualized with both ScanCap and a standard endoscope equipped with NBI. The Michelson contrast of the vessels in the ScanCap images, as compared to the background measured, was 0.38. The Michelson contrast of the vessels in the endoscopic image, as compared to the background, was 0.32.

**Figure 7:**
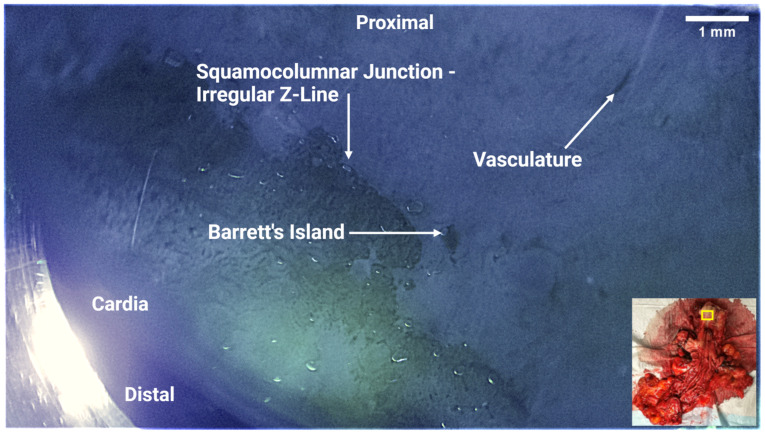
ScanCap image of resected esophageal specimen. The image shows the entire ScanCap field of view (12 mm × 7 mm). Inset, lower right: Photograph of resected specimen showing approximate location of ScanCap image acquisition (yellow rectangle). The proximal end of the esophagus specimen was on the top right, while the distal end was on the bottom left. The irregular squamocolumnar junction (Z-line) shows characteristic features of esophageal metaplasia. Barrett’s islands and dilated vasculature were also observed in the capsule images.

A resected esophageal specimen was acquired immediately post-operatively from a patient undergoing Ivor Lewis Esophagectomy for confirmed T1 esophageal adenocarcinoma. The patient had not undergone radiation therapy prior to resection. Figure 7 shows ScanCap images obtained from the esophageal specimen. The ScanCap images clearly show the characteristic features of BE and EAC. An irregular Z-line showing dysplasia from squamous esophageal tissue to granular tissue was detected, as well as a Barrett’s Island.

### 3.3. Safety Evaluation—Capsule Integrity and Potential for Esophageal Damage

After 24 h submersion in a pH 1.4 solution and inspection, no liquid was observed to have entered the capsule. Following the compressed air test, no air leakage was detected, and the pressure within the capsule remained steady, indicating that the capsule seal remained intact.

After 13 repeated passes of ScanCap through a resected porcine esophagus, no evidence of damage or abrasions was observed at any point within the esophagus or on the capsule. The capsule and tether remained intact throughout the procedure.

## 4. Discussion

This manuscript describes the development and initial evaluation of ScanCap—a high-resolution, low-cost capsule endoscopy system, designed to image the upper GI tract. ScanCap integrates BGI, high-resolution image capture, and 360-degree imaging within a 12.8 × 35.5 mm tethered capsule. ScanCap images from normal volunteer oral mucosa and ex vivo esophageal specimens demonstrate the ability to visualize irregularities at the GEJ and microvascular morphology, indicative of dysplasia. ScanCap is meant to facilitate repeated use by untrained personnel in resource-limited settings. At present, the cost of materials for the ScanCap capsule is USD 323 (with an additional USD 35 and USD 69 for the control module and display module and USD 1000 for the illumination module), considerably less than the cost of equipment for standard-of-care endoscopy. The performance of the ScanCap prototype demonstrates the potential for a non-invasive tethered capsule endoscope to image the GEJ without the need for sedation. Our preliminary results suggest that ScanCap can image features that have been reported in previous studies to be clinically relevant.

Table 2 compares the specifications of the ScanCap prototype to that of other capsule endoscopy systems available in the United States (PillCam, CapsoCam, Endocapsule 10, OMOM Capsule) as well as a recently developed system (OCT Capsule).

Unlike other commercially available capsules, ScanCap provides illumination for BGI, one of only two imaging modalities (the other being confocal laser endoscopy) satisfying the ASGE PIVI criteria (sensitivity > 90%, NPV > 98%) for ‘optical biopsy’ in BE [40]. ScanCap also offers comparable resolution to standard commercially available endoscopes, as well as the ability to capture a 360-degree side view of the esophageal mucosa with a higher resolution than other available capsules (Table 2). This, combined with the use of a tether, should provide superior coverage, as compared to other forward-facing or untethered capsule imaging technologies, which traverse the GEJ at a speed too high to properly screen for squamocolumnar metaplasia.

However, the ScanCap prototype is 35.5 mm in length, which is longer than commercially available capsules (26.00 mm to 31.00 mm). Efforts to reduce the length of the ScanCap capsule by further miniaturizing the capsule components are in progress. ScanCap is designed to be swallowed by the patient; however, the current prototype utilizes an Arducam extension cable to connect the pi camera and transmit video signal to the display module. Currently, the Arducam connector is too large to fit within the capsule shell and does not reach a sufficient length to reach the GEJ comfortably [48]. To alleviate these limitations, we are redesigning the connection component between the Arducam and pi camera.

## 5. Conclusions

ScanCap is a low-cost tethered capsule endoscope system with side-looking imaging geometry, combined with blue and green LED illumination, enabling the acquisition of BGI images, with an instantaneous FOV of 12 mm × 7 mm and resolution of 7.8 µm. With an internal rotating mirror and an image acquisition speed of 10 fps, the prototype ScanCap system is designed to image a 5 cm portion of the lower esophagus (the location where BE is formed) in less than 20 s, enabling a total procedure time of <1 min. Preliminary imaging results from resected esophageal specimens and in the oral mucosa of healthy volunteers demonstrate the ability of ScanCap to image microvascular patterns with high contrast. This implementation of BGI in a capsule endoscope format represents a step towards a low-cost BGI imaging system that could potentially be used to screen for BE in unsedated patients.

## Figures and Tables

**Table 1:** Specifications of ScanCap prototype compared to standard-of-care endoscopy system.

System	Cost	Spatial Resolution Field of View View Angle	Image Acquisition Speed	Imaging Modality	Sedation Used
ScanCap	$1427 ($323 capsule + $35 control module + $69 screen display, $1000 illumination module)	15 µm 12 mm × 7 mm 360° side viewing	10 fps	Blue Green Illumination	No
Olympus CV-190 (GIF-HQ190) [41]	$83,000	10–70 µm 100 mm × 80 mm 140° forward viewing	60 fps	White light & Narrow band imaging	Yes

**Table 2:** Comparison between ScanCap and other capsule endoscopy methods commonly used or in current development.

System	Use	Size Length × Diameter (mm)	Battery Life (h)	Image Sensor Size (Pixels)	Field of View	Communication
PillCam SB3 [43]	Small bowel	26.2 × 11.4	11	340 × 340	156° forward viewing	Radio Frequency
CapsoCam Plus [44]	Small bowel	31.0 × 11.0	15	1920 × 1080	360° side viewing	Retrieval
Endocapsule 10 [45]	Small bowel	26.0 × 11.0	12	NA	160° forward viewing	Radio Frequency
OMOM Capsule [46]	Small bowel	27.9 × 13.0	12	512 × 512	172° forward viewing	Radio Frequency
OCT Capsule [47]	Esophagus	28.0 × 12.0	NA	NA	360° side viewing	Tethered
ScanCap	Esophagus	35.5 × 12.8	NA	1920 × 1080	360° side viewing	Tethered

Abbreviations: SB = Small Bowel, OCT: Optical Coherence Tomography, NA: Not Available (not publicly disclosed).

## Data Availability

The data presented in this study are available on request from the corresponding author.
